# Douglas fir stimulates nitrification in French forest soils

**DOI:** 10.1038/s41598-019-47042-6

**Published:** 2019-07-23

**Authors:** Bernd Zeller, Arnaud Legout, Séverine Bienaimé, Bruno Gratia, Philippe Santenoise, Pascal Bonnaud, Jacques Ranger

**Affiliations:** 1 INRA Grand-EST Nancy, UR 1138 Biogéochimie des Ecosystèmes Forestiers, Route d’Amance, 54280 Champenoux, France; 2 Office National de Forêts (ONF), Unité Territorial de Darney-Bains, 6 rue des Rochottes, 88260 Darney, France

**Keywords:** Element cycles, Forestry, Forest ecology

## Abstract

Douglas fir trees presumable stimulate nitrification in the soil. We studied in 21 French Douglas fir forests if and how nitrification is modulated by soil properties, past land use and current forest management. Soil (0–10 cm depth) was collected and initial concentrations of N-NH_4_^+^ and N-NO_3_^−^, potential net nitrogen mineralization (PNM) and net nitrification (PNN) rates and microbial biomass were measured. At 11 of the 21 sites, annual nitrate fluxes in the soil were measured using anion exchange resin bags. Soils contained between 2.3 to 29.4 mg N-NO_3_^−^ kg soil^−1^. About 86% (±14%) of mineral N was nitrate. The proportion of nitrate increased to almost 100% during incubation. PNN varied from 0.10 mg N kg soil^−1^ day^−1^ to 1.05 mg N kg soil^−1^ day^−1^ (21 sites). Neither the initial nitrate concentration nor PNN was related to soil chemistry (pH, % C, %N, P, CEC), microbial biomass, texture, past land use or thinning. *In situ* net nitrate accumulation (NNA) estimated with resins beds varied from 4 to 100 kg N-NO_3_^−^ ha^−1^ yr^−1^ (11 sites). It was positively correlated with base saturation, clay content, ELLENBERG N, temperature and negatively with soil organic N, C/N ratio and precipitation.

## Introduction

Trees are an integral part of nitrogen cycling in forests because trees take up nitrogen, grow, produce litter which decompose and releases nitrogen. Tree species influence nitrogen cycling through uptake of mineral and/or organic N, litter quality and quantity and interactions with microbes at the plant root interface^1–3^. Although, availability of mineral nitrogen is still a factor limiting the productivity of forest ecosystems in many biomes^4–6^. Especially, tree species with a high biomass increment and thus, a high demand for nitrogen and other nutrients, as Douglas fir (*Pseudotsuga menziesii (Mirb.)Franco var. menziesii)*) need to apply/develop efficient strategies to match these requirements.

Since the late 50’s until the 80′s significant areas in France were afforested with Douglas fir while during the same period broadleaf forests were converted into Douglas fir forests. Under optimal conditions annual increment of Douglas fir may reach 30 m^3^ ha^−1^ yr^−1^ which rank this species among the most productive coniferous species in Europe^7,8^. Since its discovery by the Scottish botanist David Douglas in 1824 this forest tree species had been introduced in Europe and contributes now to about 3% or 421000 ha of the French forest cover^7,9,10^. The most commonly cited reasons for this success are (i) rapid initial growth and high production levels^11^ over a wide range of environments through effective coping strategies^12^, (ii) efficient nutrient uptake strategies and biomass production^13^, (iii) absence of major pests and pathogens^10^ and at last (iv) wood products with rheological qualities that do not deteriorate with the radial growth rate^14^.

The impact of Douglas fir on biogeochemical cycles have been extensively studied since the 1960’s in its native range^15–18^ or when introduced as alien species in Europe^19^. In Europe, some studies focused on its impact on the nitrogen cycle^20–23^. Among them, Jussy *et al*. at Vauxrenard (France) and Zeller *et al*. at Breuil (France), highlighted the dominance of nitrate as main mineral N form in the topsoil when this species was planted on previous farmland or broadleaf forest^24,25^. At both sites, soils were acid, nutrient poor with low amounts of plant available Ca and Mg. Such soils are representative for about half of the area of French Douglas fir forests^26^. In addition, in a common garden experiment with 6 tree species, a fairly rapid and significant increase of the nitrate concentration in soil cores transferred 18 months earlier from 3 low nitrifying tree species into a Douglas fir stand happened^27^. Based on this experiment Andrianarisoa *et al*. suggested that Douglas fir triggers nitrification through interactions with nitrifying bacteria at the root – soil interface^28^. Although the precise mechanism(s) by which Douglas fir may stimulate nitrification and enhance soil nitrate concentrations remain still unknown, as well as a possible modulation by environmental factors (e.g. soil properties, past land use, current forest management).

Consequences of high soil nitrate concentrations and resulting leaching of nitrate in Douglas fir forests is loss of cations (Al, Ca, Mg) and soil acidification^19,29^. However, this impact depends on soil conditions e.g. under acidic conditions when nitrate is not immobilized probably in relation to deficient microbial activity or root uptake, a massive leaching of nitrate and monomeric Al^3+^ occurred in deep drainage solutions^30^. Such a phenomenon potentially affects the quality of surface waters^31^.

Up to now, almost all studies about nitrate concentrations and potential nitrification were obtained in Douglas fir forests on acid, more or less nutrient poor soils. Thus, the question arises if this tree species affects soil nitrate concentrations and potential nitrification in a generalized way in less acid and more fertile soils. Our hypothesis is that in Douglas fir forest soils in France, nitrate is the dominant mineral N form and that potential nitrification rates were modulated by soil properties. In the light of this hypothesis our objectives were to (1) evaluate the amount and form of mineral N in the mineral soil (0–10 cm depth) in 21 Douglas fir forests in France, distributed all over the distribution area of this species, and assess net mineralization and nitrification potentials under controlled conditions, (2) compare these potentials to their expression under field conditions using ion-exchange resins and (3) tackle how environmental factors (e.g. climate, soil fertility, stand structure, tree growth and past land use) may modulate the availability of mineral nitrogen in the soil of Douglas fir forests.

## Results

### Soil chemical status of the experimental sites

Amongst the studied sites, soil types and their soil physico-chemical characteristics varied broadly. Cambisols formed the biggest group, the other soil types like Podzols, Alfisols, Andosols or Planosols are less abundant (1–2 sites per soil type) (Table 1). In line with the soil type, soil pH, concentrations of soil organic C and soil organic N differed between the sites but remained in a range commonly observed in French forest soils. As for the soil type, the texture of the soils covered a large range, from sandy to loamy until clayey soils. Most other variables as, CEC, exchangeable cations like Al and Ca showed a similar pattern than the former parameters thus validating the selection of each site. Indeed, sites were thought to represent the whole panel of French soils now covered by Douglas fir forests.

**Table 1:** Soil type and soil chemical characteristics of the studied sites (soils 0–10 cm). Ca^2+^, Mg^2+^, K^+^  = exchangeable Cations (mg g^−1^), CEC = cation exchange capacity (meq. 100 g^−1^), S/T = base saturation (%), Al^3+^ (g kg^−1^) and phosphorus as P_2_O_5_ (g kg^−1^). In bold and *, sites (n = 11) where soil humidity, soil solution chemistry and nutrient fluxes are measured (solutions are collected once per month).

Site	Soil type	C (%)	N (%)	pH	Sand (%)	Silt (%)	Clay (%)	Ca^2+^ (mg g^−1^)	Mg^2+^	K^+^	CEC (meq. 100 g^−1^)	S/T	Al^3+^	P_2_O_5_
ADI	Hyperdystric Cambisol	3.45	0.23	4.45										
ALL*	Andosols	10.10	0.72	4.67	34.9	46.0	19.1	3.30	0.58	0.43	9.47	53.1	17.3	1.13
BOU	Alfisols	3.43	0.17	3.86	23.5	63.2	13.3	1.16	0.31	0.17	5.97	35.9	1.2	0.09
CONT*	Eutric Cambisol	2.80	0.20	4.36	8.1	74.3	17.6	2.98	0.53	0.51	7.98	58	1.7	1.10
DOU34	Dystric Cambisol	4.01	0.33	4.56	22.9	34.9	31.2	2.46	0.43	0.16	5.36	56.9	24.0	0.03
GAI*	Dystric Cambisol	4.01	0.27	3.94	10.7	70.8	19.9	0.75	0.25	0.35	8.38	23.9	2.3	0.39
MEY*	Entic Podzols	9.40	0.66	3.96	52.2	23.7	24.1	0.09	0.13	0.27	9.90	10.7	3.9	0.21
MIG	Dystric Planosol	3.85	0.25	5.24	21.4	53.7	24.9	1.06	0.19	0.32	5.62	35.2	2.3	0.11
MONT*	Eutric Planosol	2.46	0.22	4.44	13.8	60.6	25.6	2.06	0.32	0.41	7.30	57.3	6.1	0.63
OLL*	Hyperdystric Cambisol	3.39	0.29	4.12	55.9	25.1	19.0	0.48	0.13	0.17	6.35	15.5	2.9	0.27
ORL-T*	Entic Podzols	10.98	0.74	4.13	62.0	21.7	16.3	0.09	0.13	0.28	8.02	9.9	6.5	0.20
ORL-F*	Entic Podzols	7.69	0.48	4.32	76.1	12.8	12.6	0.28	0.12	0.16	5.86	4.6	6.9	0.15
PAU*	Hyperdystric Cambisol	4.70	0.37	4.20	52.5	25.5	22.0	1.11	0.31	0.32	9.63	27.2	4.1	1.15
PEY-T	Entic Podzols	5.30	0.36	4.09	61.0	18.2	20.8	0.42	0.21	0.34	11.52	12.8	4.3	0.12
PEY-ESC	Entic Podzols	5.52	0.36	4.00	70.4	15.4	14.2	0.21	0.74	0.23	5.10	13.3	4.2	0.15
PIQ	Hyperdystric Cambisol	6.74	0.49	4.18	63.3	20.4	16.6	0.26	0.75	0.15	4.50	13.5	7.8	0.46
ROQ*	Eutric Cambisol	4.31	0.37	4.20	25.1	47.9	27.0	7.62	1.16	0.49	14.03	70.0	1.1	0.13
SAL-B	Calcaric Cambisol	10.08	0.70	6.71	24.7	16.8	58.5	32.2	4.14	0.86	40.21	96.4	2.9	0.67
SAL-H	Andosols	5.41	0.38	4.81	17.6	52.6	29.8	8.57	2.72	0.7	12.90	94.4	3.8	0.62
VIL*	Eutric Cambisol	2.58	0.19	4.04	14.1	54.6	31.3	1.72	0.34	0.39	8.92	35.7	4.4	0.14
VULC	Andosols	6.42	0.47	5.35	35.8	43.1	21.1	1.07	0.19	0.17	3.99	39.5	24.1	0.63

### Initial mineral N content

In the 84 soil samples (0–10 cm layer), collected at the 21 sites, the soil pH covered a range from 3.8 to 6.7, the C concentration varied from 2.5% C to 10.9% C, the N concentration varied from 0.2% N to 0.7% N and the C/N varied from 11.0 to 20.1 (Table 1). In the field fresh soils, immediately extracted after their sampling in the Douglas fir forests, the ammonium concentration varied from 0.7 to 12.8 mg N-NH_4_^+^ kg soil^−1^ and the nitrate concentration varied from 2.3 to 29.4 mg N-NO_3_^−^ kg soil^−1^ (Table 2). In all soils, the percentage of nitrate varied from 56% to 93% with 86% (±14%) as a mean. Nitrate was always much more abundant than ammonium at all sites.

**Table 2:** Initial nitrate concentration at T = 0 and percentage of nitrate in field fresh soils (n = 4), potential net nitrification rate (PNN) and percentage of nitrate in incubated soils (n = 4), net nitrate accumulation rate (NNA) measured with resin beds in the field (n = 30). Mean values and standard variation. In bold and *, sites (n = 11) where soil humidity, soil solution chemistry and nutrient fluxes are measured.

Site	T = 0 (mg N-NO_3_^−^ kg soil)	% (N-NO_3_^−^)	PNN (mg N-NO_3_^−^ kg soil^−1^ day^−1^)	% (N-NO_3_^−^)	NNA (kg N-NO_3_^−^ ha^−1^ yr^−1^)
ADI	22,18 ( ± 10,25)	88	0,37 ( ± 0,22)	95	
ALL*	13,36 ( ± 6,27)	93	1,05 ( ± 0,45)	100	25,79 ( ± 5,12)
BOU	5,78 ( ± 1,90)	82	0,37 ( ± 0,14)	99	
CONT*	19,99 ( ± 6,16)	99	0,69 ( ± 0,25)	100	36,39 ( ± 8,13)
DOU34	7,90 ( ± 1,81)	95	0,62 ( ± 0,29)	98	
GAI*	10,30 ( ± 0,80)	75	0,63 ( ± 0,21)	98	42,09 ( ± 11,20)
MEY*	4,03 ( ± 1,37)	62	0,19 ( ± 0,11)	53	4,30 ( ± 2,27)
MIG	10,95 ( ± 1,87)	85	0,83 ( ± 0,10)	91	
MONT*	29,44 ( ± 12,44)	99	0,44 ( ± 0,12)	99	78,18 ( ± 14,55)
OLL*	3,10 ( ± 1,59)	80	0,38 ( ± 0,11)	94	28,87 ( ± 10,38)
ORL-F*	2,31 ( ± 0,91)	80	0,64 ( ± 0,13)	96	10,53 ( ± 3,00)
ORL-T*	7,39 ( ± 1,76)	78	0,81 ( ± 0,07)	99	18,53 ( ± 4,95)
PAU*	6,75 ( ± 3,22)	94	0,60 ( ± 0,20)	99	37,86 ( ± 12,05)
PEY-F	10,60 ( ± 6,25)	96	0,58 ( ± 0,15)	98	
PEY-T	11,93 ( ± 1,48)	91	0,53 ( ± 0,19)	99	
PIQ	12,93 ( ± 2,02)	92	0,47 ( ± 0,04)	98	
ROQ*	10,30 ( ± 8,13)	82	0,29 ( ± 0,05)	99	101,26 ( ± 23,24)
SAL-B	4,07 ( ± 0,73)	53	0,10 ( ± 0,15)	83	
SAL-H	27,32 ( ± 12,81)	95	0,68 ( ± 0,12)	100	
VIL*	10,66 ( ± 5,38)	84	0,34 ( ± 0,12)	98	63,15 ( ± 12,24)
VULC	19,05 ( ± 7,46)	98	0,66 ( ± 0,27)	99	

### Potential net mineralization (PNM) and potential net nitrification (PNN) rate

**Figure 1:**
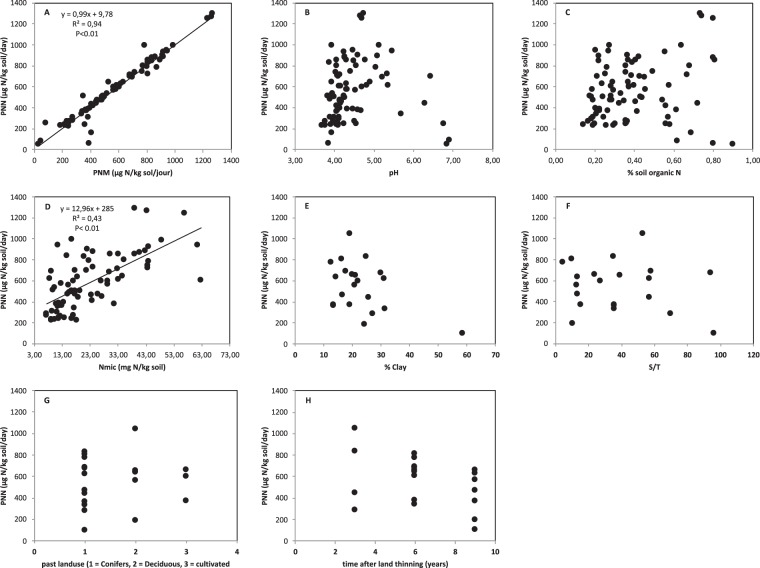
Relationships between potential net nitrification (PNN) and (**A**) potential net mineralization (PNM), (**B**) pH, (**C**) soil organic N, (**D**) microbial biomass N (except MEY and SAL_B), (**E**) clay content, (**F**) base saturation (S/T), (**G**) past land use (1 = conifers, 2 = broadleaf, 3 = pasture or farmland) and (**H**) forest management (years since last thinning).

The daily PNN rate varied by a factor of 10 among the sites, from 0.10 mg N-NO_3_^−^ kg^−1^ day^−1^ (SAL-B) till 1.05 mg N-NO_3_^−^ kg^−1^ day^−1^ (ALL) (Table 2). After three weeks and at the end of the incubation period of six weeks, about 90–98% of mineral N was present as nitrate, except at one site (53% at MEY). We observed a highly significant linear relationship between the rates of PNM and PNN (r^2^ = 0.97, P < 0.05) (Fig. 1A). The few values, observed below the 1:1 line, all correspond to the same site (MEY) with the lowest percentage of nitrate. Both, PNM and PNN varied among the 21 studied sites, but PNN was neither related to the soil organic N concentration, the pH, the base saturation (S/T ratio) or the clay content (Fig. 1B, C, E, F). Neither a trend nor any relationship appeared between the PNN and other site-specific variables like former land use and current forest management (Fig. 1G, H). Obviously, the species effect on PNN rates is much stronger than site-specific parameters as soil fertility or past land use. Exclusion of the data from the two sites with the lowest PNN and the lowest or highest pH (MEY and SAL-B) revealed a positive correlation between PNN and soil microbial biomass C and N (r^2^ = 0.43, P < 0.05) (Fig. 1D).

### *In situ* net nitrate accumulation (resin beds)

**Figure 2:**
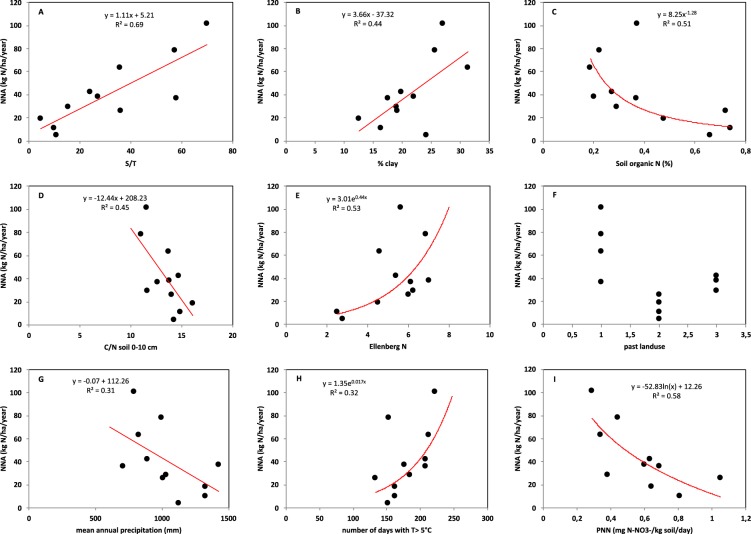
Relationships between annual net nitrate accumulation (NNA) and (**A**) base saturation (S/T), (**B**) clay content, (**C**) soil organic N, (**D**) C/N of the 0–10 cm soil), (**E**) Ellenberg N, (**F**) past land use, (**G**) mean annual precipitation, (**H**) number of days with T > 5 °C and (**I**) PNN (except Mey). Data obtained at 11 sites.

As for PNN, a large variation of nitrate accumulation in resins was observed among the 11 sites (Table 2). Average annual net nitrate accumulation (NNA) varied by a factor of 25 among the sites, from 4.3 kg N-NO_3_ ha^−1^ yr^−1^ at MEY to 101.3 kg N-NO_3_ ha^−1^ yr^−1^ at ROQ. Nevertheless, beside these two extremes, NNA varied in a narrower range among the remaining 9 sites (10.5 kg N-NO_3_ ha^−1^ yr^−1^ to 78.2 kg N-NO_3_ ha^−1^ yr^−1^). NNA was positively correlated to the base saturation (S/T ratio, r^2^ = 0.69; P < 0.05) (Fig. 2A), the clay content (r^2^ = 0.44; P < 0.05) (Fig. 2B), the Ellenberg N (r^2^ = 0.53; P < 0.05, Fig. 2E) and the number of days with a temperature >5 °C (r^2^ = 0.32; P < 0.05, Fig. 2H). NNA was negatively correlated to soil organic N (r^2^ = 0.51; P < 0.05) (Fig. 2C), the C/N ratio of the mineral soil 0–10 cm (r^2^ = 0.45; P < 0.05) (Fig. 2D) and mean annual precipitation (r^2^ = 0.31; P < 0.05) (Fig. 2G). NNA was higher in former coniferous forests than in former broadleaf forests and former farmland or pasture (Fig. 2F). Excluding the data from the site MEY, with the lowest PNN and NNA, revealed a negative relationship between NNA and PNN (r^2^ = 0.58; P < 0.05) (Fig. 2I).

### Multivariate analysis

**Figure 3:**
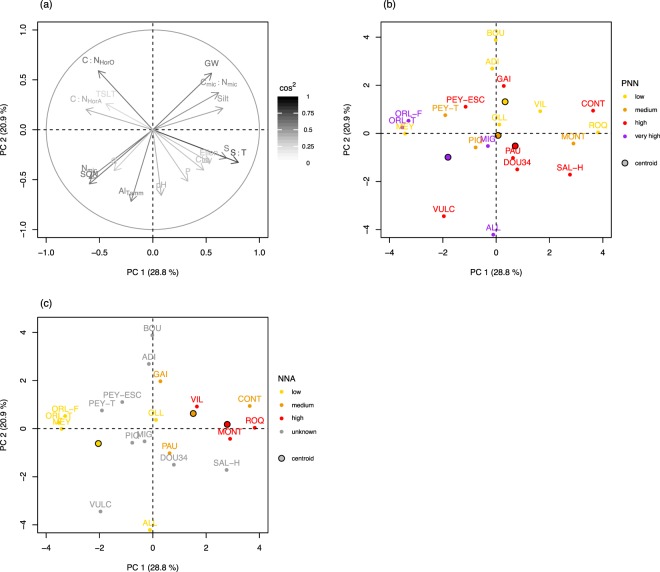
(**a**) distribution of the 17 parameters (soil chemistry, stand characteristics) (**b**) distribution of the sites according to the PNN group, (**c**) distribution of the sites according to the NNA group.

A Principal Component Analysis (PCA) has been used to summarize the potential relationship between site characteristics (soil and stand properties) and net nitrification (PNN) or net nitrate accumulation rate (NNA)(Fig. 3, Table S1). Base saturation (S/T), sum of base cations (S), silt and C_mic_/N_mic_ are opposed to C/N_horA_, N_mic_ and soil organic nitrogen (SON) on the principal component 1 (PC1). C/N_horO_, duration of the growing season (GW) are opposed to aluminum extracted by the Tamm method (Al_Tamm_), pH, soil organic nitrogen (SON) and phosphorus (P) on the principal component 2 (PC2). The principal plan PC1-PC2 did not allow to discriminate the PNN groups, whereas at least the high NNA group differentiates from the others NNA groups. These findings corroborate previous results presented in Figs 1, 2, that emphasized strong relationships between net nitrate accumulation rate and some site characteristics, whereas no clear relationship was observed between net nitrification and site characteristics.

### Review of PNN and PNM in coniferous, broadleaf and Douglas fir forest soils

**Figure 4:**
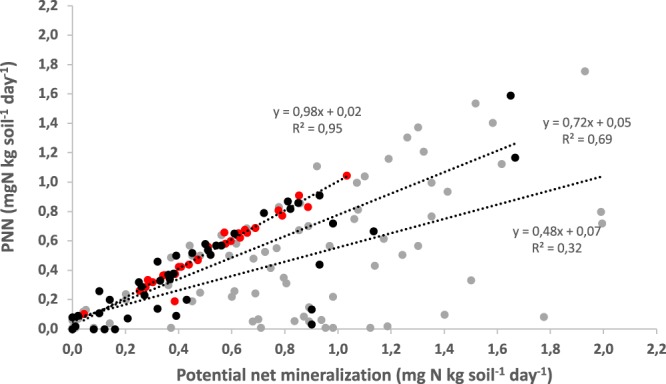
Relationships between PNM and PNN in French Douglas fir forests soils (red dots), in coniferous forests soils (black dots) (White *et al*., 1988; Gower and Son 1992; Menyailo *et al*., 2002; Nugroho *et al*., 2006; Colman and Schimel 2013; Urukawa *et al*., 2016) and in broadleaf forest soils (grey dots) (Morris and Boerner 1998; Verchot *et al*., 2001; Gower and Son; Menyailo *et al*., 2002; Andrianarisoa *et al*., 2009; Colman and Schimel 2013; Urukawa *et al*., 2016)

In the selected studies (n = 9), PNM and PNN rates were obtained by incubating mineral soils under fairly similar incubation conditions than ours (temperature, soil water content). Over the panel of published data, large variations in PNM and PNN among coniferous, broadleaf and Douglas fir forests were observed (Fig. 4). In Douglas fir forests, there is a strong positive correlation between PNM and PNN (r^2^ = 0.95, p < 0.05) which follows the 1:1 line, indicating that PNN = PNM. A similar positive correlation is observed for coniferous forests (r^2^ = 0.69, p < 0.05). Compared to Douglas fir, PNN is lower than PNM in coniferous forests, but nitrate is still the dominant mineral N form. Although a large variability, in broadleaf forests, a positive relation between PNN and PNM is observed (r^2^ = 0.32, p < 0.05). Compared to Douglas fir and coniferous forests, PNN is much lower than PNM in broadleaf forests. Mean rates of PNM are 0.54 mg N kg^−1^ day^−1^, 0.48 mg N kg^−1^ day^−1^ and 0.84 mg N kg^−1^ day^−1^ in Douglas fir forests, coniferous forests and broadleaf forest, respectively. Mean rates of PNN are 0.55 mg N-NO_3_^−^ kg^−1^ day^−1^, 0.40 mg N-NO_3_^−^ kg^−1^ day^−1^ and 0.48 mg N-NO_3_^−^ kg^−1^ day^−1^ in Douglas fir forests, coniferous forests and broadleaf forest, respectively.

## Discussion

### Dominance of nitrate in the topsoil

Our hypothesis that nitrification is predominant in French Douglas fir forests soils is supported by the initial ammonium and nitrate concentrations measured in the topsoil at the 21 sites.

Firstly, although large inter-site variations of the initial soil nitrate concentration (3–30 mg N kg soil^−1^) were observed, we show that a dominant share of the mineral N in the 0–10 cm soil (on average 86% ± 14%) was present as nitrate. As this percentage was neither related to soil properties nor forest management, we suppose that the main reason for such an imbalance between ammonium and nitrate is the tree species itself. Climate events (drought or enhanced soil temperature) may reinforce or weaken the amount of residual soil nitrate but this factor will not basically modify this trait. In addition, the average autumnal nitrate concentration in Douglas fir (12 mg N-NO_3_^−^ kg soil^−1^) is definitively at the upper boundary compared to nitrate concentrations measured in other temperate forests soils^25,32,33^ and the Ellenberg N values of the forest floor vegetation confirms the long-lasting dominance of nitrate in the studied Douglas fir forests. Usually, the leftover of nitrate in late autumn is a sign that the production of nitrate exceeds the uptake by the trees and the understory vegetation^34^. Our results are consistent with earlier findings at the Breuil common garden experiment^27,30,^^35^, where apparently higher soil and soil solution nitrate concentrations were measured in Douglas fir compared to other deciduous or coniferous species. In addition, Douglas fir increased rapidly the soil nitrate concentration in soil cores transferred into the Douglas fir plots from other less nitrifying plots whereas the opposite effect was observed in soil cores from Douglas fir transferred into less nitrifying plots^27^.

Secondly, we show that the potential net nitrification (PNN) is high at all sites, except one and is probably only limited by the bottleneck’s depolymerization of soil organic N and ammonification. These findings illustrate the high potential of nitrifying bacteria to convert ammonium into nitrate and corroborate the dominance of nitrate in French Douglas fir soils. We may suppose that under non-limiting conditions, the potential nitrification rate would be even higher than observed. Positive or negative effects of different tree species on net and gross N mineralization and nitrification had been observed in a certain number of common gardens experiments^36–40^. In the above-mentioned studies, net mineralization was close to net nitrification in Douglas fir soils, a factor that approves the dominance of nitrate under this species. Nevertheless, old growth Douglas fir forests in the US did not really fit into this schema, because net nitrification is here much lower^41^. In those older forests the buildup of thicker humus layers indicates modifications of the soil chemistry and reduction of biological activity with a switch towards a higher abundance of fungi. For instance, increase in forest floor thickness along a chronosequence of beech stands resulted in a sharp decrease in nitrification^42^.

### Modulation of soil nitrate contents in Douglas fir forests

Litter (needle and roots) is the most important source of recent soil organic matter in the studied Douglas fir forests and the thin organic layers in all studied stands indicated a highly efficient team constituted by fauna, fungi and bacteria that decomposes litter thus creating a continuous flux of soil organic matter into the topsoil. Some authors also reported a positive effect of Douglas fir on the humus C:N ratio, soil pH and base saturation^10,29^. Nevertheless, the absence of any relation between PNN and soil organic C and N suggest that soil organic matter is probably not a principal driver of nitrification at our sites. Moreover, the potential net mineralization and net nitrification measured at the 21 sites cope fairly little with soil chemical parameters. These findings concur with the results of two meta-analysis with numerous tree species^43,44^ where a minor part of the observed variability of net mineralization and nitrification was explained by climatic and soil chemical parameters (33% by MAP + %C + %N + clay content) but both studies supposed that biological parameters like soil organic matter quality and microbial community composition strongly increase this percentage.

Nitrification had also been related to the tree species^39,45,46^, past land use^32^ and forest management practices^18,29,47–49^. In the present study, recognized drivers of nitrification in forest ecosystems like past land use and forest management practices did not mark net nitrification and nitrate accumulation in resins in the Douglas fir stands. Thus, other intrinsic parameters seem responsible for the predominance of nitrate in French Douglas fir soils. Different kinds of interactions at different levels (molecular, diversity) between the tree species and microbes and/or soil parameters are appropriate to affect soil N transformations and nitrification^50,51^.

Some tree species like Norway spruce or birch produce monoterpenes or diterpenes as agents to down regulate nitrifying bacteria^52–54^ while the absence of monoterpenes was observed in Douglas fir stands^52^. Moreover, Nugroho *et al*. suggested that Douglas fir could even up regulate the number of nitrifying bacteria: although the mechanism is not well understood, a positive correlation between AOB’s like *Nitrosospira* and nitrate production was observed^39^. Andrianarisoa *et al*. revealed that interactions at the root-soil-interface leading to a rapid up regulation of the number of ammonium oxidizing bacteria (AOB) in soils cores transferred from two low nitrifying species (Norway spruce, Nordmann fir) into a Douglas fir plot^28^. According to these studies there is some evidence that Douglas fir stimulate AOB’s, but how is still an open question. Nugroho *et al*. supposed that tree species regulate AOB’s through their effects on the soil C/N ratio, but Zeller *et al*. observed a clear difference between different species in net and gross nitrification in a common garden experiment where soils had a similar C/N ratio^25,39^. As previously detailed, neither the humus nor the soil C/N ratio was a primary determinant of PNN or NNA in our study. As at our sites climate and soil chemistry varied largely, the high PNN and soil nitrate concentrations suggests a straight stimulating effect of Douglas fir on nitrifying bacteria^39^. In addition, Douglas fir is a neophyte in Europe, young trees were often inoculated with north American mycorrhizal strains as Laccaria laccata S238N^55^. Probably, Douglas fir fine roots are not familiar to most of the ectomycorrhizal fungi (ECM) present in European forest soils which may affect the interactions between microorganisms and fine roots. In the light of such a scenario it could be possible that with ongoing time the ectomycorrhizal diversity increases thus inducing a switch towards a more conservative N cycling.

### *Ex situ* versus *in situ* approach and implication for forest management

Ion exchange resins (IER) have been recognized as a relevant tool for the assessment of the availability of mineral nitrogen in soils^56^. IER have the big advantage to investigate under real field conditions (temperature, moisture, soil structure, biotic factors) soil mineral nitrogen fluxes with a very limited initial soil disturbance^57^. The availability of mineral nitrogen in the nitrate form, as measured by the resins bags is the expression of the difference between the production by nitrifying bacteria, the immobilization by microorganisms and the vegetation and finally loss by leaching of excess nitrate. The negative correlation between NNA and PNN reflects the complexity of the N cycle in Douglas fir forests and underlines that the potential is not expressed *in situ*. Contrary to PNN, NNA was related to the S/T ratio, the clay content and the soil organic N concentration. Interestingly, less nitrate was accumulated in soils with higher soil organic C and N contents, which is somewhat surprising and opposite to observations made in other forests^34^. Sites characterized by higher soil fertility (S/T ratio, Ca concentration) sustained nitrification but also loss of nitrate. Such a behavior suggest/indicate that even under conditions of optimal growth (nutrients, water) for Douglas fir a substantial proportion of nitrate is probably transferred into deeper soil layers and/or lost to the ground water^58^. The greater amount of clay at these sites could result in more fine porosity, which would be a suitable habitat for nitrification for much of the year. Leaching of excess nitrate will result in loss of cations, acidification and *in fine* deterioration of drinking water quality^29–31,59^. At least, two forest management measures could reduce nitrate leaching: mixed stands composed of beech and Douglas fir^29,60^ and fertilization with Ca -Mg –P for acid nutrient poor soils, because such a treatment reduced the nitrate concentration significantly in soil solutions collected at 0.6 m soil depth in a so treated Douglas fir stand^35^. Nevertheless, further experiments are necessary to evaluate the impact of fertilization on soil nitrate fluxes in other acid contexts and along a soil fertility gradient.

## Conclusion

All results converge to the same conclusion, that namely Douglas fir, an alien tree species in Europe, has a strong encouraging/stimulating effect on nitrification and top soil N-NO_3_^−^ concentrations either *in situ* and *ex situ*. This effect comes first into sight in the field fresh soil samples (collected in autumn) from all 21 sites. Potential net nitrification rates (PNN) in incubated soils but also the net nitrate accumulation (NNA) in the field corroborate the domination of nitrate in French Douglas fir soils. Net nitrate accumulation is higher in more fertile soils and at warmer sites. This suggests that even under optimal conditions for tree growth (high biomass increment) an excess of nitrate remains in the soil with a peak in autumn. As nitrate is highly mobile in the soil profile, leaching loss of nitrate and cations may affect surface and groundwater quality, as well as the sustainability of soils by an acidification process. Future studies are needed to assess the environmental impact of Douglas fir by evaluating properly the leaching losses of nitrate at the plot scale and at larger scales (watersheds etc.) in monospecific Douglas fir forests. Another perspective, which seems the most difficult, is to understand the mechanism(s) by which Douglas fir stimulates the activity of nitrifying bacteria. Lastly, further research should focus on how forest management measures may mitigate nitrate fluxes, probably by the set up of mixed stands and through liming/fertilization.

## Material and Methods

### Sites

All over France, 21 Douglas fir forests (private and public forests) were selected from a larger number of sites (Table 3, Fig. S1). Those sites are thought to represent all areas in France where Douglas fir had been introduced since the last century and where Douglas fir covers a significant surface of the forested area. Soil properties (physical and chemical parameters) were one important criterion for the final selection of a site in order to represent the whole panel of soil types and soil fertility. Additional criteria were vegetation cover (Ellenberg N) past land use and current forest management practices^61^. Climatic parameters like annual rainfall, air temperature and length of the growing season were recorded but not included into the site selection process. Among these 21 sites, a sub-network of 11 sites was set up with the objective to monitor continuously soil moisture, fluxes of nutrients and litter fall.

**Table 3:** Description of the study sites (mean annual precipitation, altitude, age and number of the trees, past land use, G = basal area, SV = days with a daily mean temperature > 5,8 °C, Ellenberg N = indicator of N availability). Sites in bold and with * belongs to the network of sub-sites with continuous monitoring of soil solution chemistry, soil humidity and litterfall.

Site	Code	Precipitation (mm per year)	Altitude (m)	SV (days)	Age (years)	Trees per ha	G (m^2^ ha^−1^)	Ellenberg (N)	Past land use
Adinfert	ADI	681	117	208	37	374	34	4.2	Coniferous Forest (CF)
Allagnat*	ALL*	917	1005	133	68	211	53	6.0	Deciduous forest or heather (DF)
Bouillancourt	BOU	780	146	211	43	368	28	4.3	Coniferous Forest (CF)
Wailly*	CONT*	706	111	208	42	204	36	6.1	Coniferous Forest (CF)
Verreries de Moussans	DOU34	1150	685	221	41	416	46	5.6	Coniferous Forest (CF)
Gaillefontaine*	GAI*	888	232	208	32	230	39	5.4	Farmland (pasture or field) (FL)
Meymac*	MEY*	1125	916	152	51	200	18	2.7	Deciduous forest or heather (DF)
Azy-le-Vif	MIG	730	302	152	38	278	14	5.9	Coniferous Forest (CF)
Montapas*	MONT*	996	288	153	46	472	36	6.9	Coniferous Forest (CF)
Olliergues*	OLL*	1030	643	185	31	450	37	6.2	Farmland (pasture or field) (FL)
Pérols sur Vézère*	ORL-T*	1326	840	162	48	429	46	2.5	Deciduous forest or heather (DF)
Pérols sur Vézère*	ORL-F*	1326	841	162	48	425	52	4.5	Deciduous forest or heather (DF)
Anglès*	PAU*	1424	802	176	41	300	39	7.0	Farmland (pasture or field) (FL)
Saint Amans le Petit	PEY-T	1250	586	172	43	1333	53	4.5	Deciduous forest or heather (DF)
Saint Amans le Petit	PEY-ESC	1250	586	172	43	285	40	4.5	Deciduous forest or heather (DF)
Le Bez	PIQ	1000	649	184	73	217	51	6.2	Coniferous Forest (CF)
Montreuil en Auge*	ROQ*	788	79	222	40	304	42	5.6	Coniferous Forest (CF)
Sallèdes	SAL-B	680	743	201	42	388	36	4.8	Coniferous Forest (CF)
Sallèdes	SAL-H	720	817	196	42	353	33	4.2	Coniferous Forest (CF)
Felleries*	VIL*	822	195	213	40	338	41	4.6	Coniferous Forest (CF)
Ceyssat	VULC	900	959	134	71	201	53	5.7	Deciduous forest or heather (DF)

### Potential net N-mineralization and nitrification rates

At each of the 21 sites, mineral soil samples (0–10 cm depth) were collected from 4 random subplots situated at 0°, 90°, 180° and 270° about 3 m around an anchor point. The more or less thin humus layer was carefully removed, then all soil (0–10 cm depth) was collected from a surface of 0.25 m^2^, put into a big box, passed through a 4 mm sieve, mixed and finally a representative sample of about 3 kg soil per subplot was shipped in a cooled container to the laboratory. Mineral N concentrations were measured in all soil samples immediately after their arrival in the laboratory. Then, the soils were stored at 4 °C, until the soil sampling campaign was completed and all soils were available for the laboratory incubation. This short storage period (3 weeks) was necessary in order to setup and start the incubation experiment with all soil samples at the same time. Potential net mineralization and nitrification rates were obtained at 20 °C and 60% water holding capacity (WHC). To meet the later criteria for each site the water holding capacity was measured in all soils before the set-up of the incubations and, if necessary, deionized water was added. Soil from each of the four subplots per site (300 g) were put into 2L Mason jars, pre – incubated during 10 days, followed by an incubation period of 42 days. During the whole period, jars were opened twice a week for about 15 minutes, the soil mixed with a spoon to assist the evacuation of accumulated CO_2_. Subsamples were collected from each jar at the end of the pre – incubation period (T0) and after 21 days (T1) and 42 days (T2) of incubation. Each sample of 40 g of soil was extracted with 200 ml of 0.5 M K_2_SO_4_, shaken during 1 hour, filtered and finally the concentrations of N-NH_4_^+^ and N-NO_3_^−^ in the extracts were measured using continuous flow analysis (SKALAR San +  + ). Potential net N mineralization (PNM = N_min_ final − N_min_ initial) was the amount of total inorganic N accumulated during the incubation period and PNN (PNN = N-NO_3_^−^ final – N-NO_3_^−^ initial) was the amount of N-NO_3_^−^ formed from the nitrification of the NH_4_^+^ already present in the soil core before the incubation as well as that mineralized during the incubation. Both were calculated as mg N kg^−1^ soil d^−1^. All concentrations and rates are presented on a dry weight basis. Microbial biomass C and N was measured after 3 weeks of incubation using the fumigation extraction method. Concentration of soil organic C and N were measured using a CHN analyzer (Carlo Erba). In addition, basic soil parameters like soil texture, pH, concentration of P and the CEC were measured at the soil analysis laboratory (LAS) of INRA in Arras.

### *In situ* accumulation of ammonium and nitrate (resin bed method)

In order to evaluate the *in-situ* N mineralization and nitrification we set up a batch of ion exchange resin traps (anionic and cationic) at 10 cm soil depth and measured over one year at 4, 8, and 12 months the amounts of ammonium and nitrate captured by the resins.

DOWEX 21 K (MERCK- CEC = 1.95 meq g^−1^ dry resin, 20–50 mesh) were used to catch anions (nitrate and others) Anionic resins were first rinsed under a slow stream of distilled water and then saturated with Cl^−^ from 1 M HCl (hydrochloric acid) at a ratio of 1000 ml HCl per 100 g of dry resin. Then, 20 grams of wet resin representing about 36–40 meq for anionic were introduced into traps made of PVC rings (Ø 50 mm and 10 mm in height), closed, from both sides by a nylon mesh (20 µm mean mesh size), allowing free percolation of water. At each of the 11 sub-sites, 6 sets of 10 resin traps were set up (total = 660 traps). After opening a pit of about 3 m long, 0,4 m large and 0,15 m deep, the traps were carefully introduced horizontally into the undisturbed soil (at a depth of 10 cm and with a minimum distance of 25 cm between two traps). Each trap was marked with dyed rope and the trench carefully refilled with the excavated soil, slight compacted and finally the organic layer was restored. In total 6 independent pits were set up per site and two pits were sampled every four months between June 2012 and June 2013.

Each resin trap was first rinsed three times with distilled water (10 ml applied with a syringe) for eliminating soil particles. Then each resin trap was weighed and stored at 4 °C until extraction. N-NO_3_^−^ was extracted with 1M NaCl at a ratio of 4 g resin per 40 ml of NaCl, after a one-hour batch contact on a rotary shaker. Concentration of N-NO_3_^−^ in the desorbed extracts was measured by colorimetry using a continuous flow analyzer (SKALAR San +  + ). Net nitrate accumulation rate (NNA) measured by resins is the amount of NO_3_–^−^N fixed by the resins during the time of incubation in the field, divided by the number of days between installation and collection. We computed only the mean annual rates of NNA. Net nitrate accumulation was expressed as kg N-NO_3_^−^ ha^−1^ y^−1^.

### Statistics

Single and multiple regression analysis were used to detect significant relationship among potential net nitrification and soil and forest management parameters. The same approach was used for the net nitrate accumulation rate. A standardized Principal Component Analysis (sPCA) has been used to analyze the data collected from the 21 sites. This data comprised 17 quantitative variables based on soil physical and chemical properties (*pH*, *silt*, *Clay*, *SOC*, *SON*, *N*_*mic*_, *C*_*mic*_:*N*_*mic*_, *S*, *S:T*, *Al*_*Tamm*_, *P*, *C*:*N*_*HorO*_, *C:N*_*HorA*_) and site characteristics (*Ellen*, *GW*, *G*, *TSLT*). The global idea of sPCA method is to project the standardized data onto a lower dimensional linear space generated by uncorrelated variables, named principal components (PC), such as the variance of the projected data is maximized. The first step is to select a minimal number of PCs while keeping a large part of the explained variance in the new PC subspace. For this, we decided (1) to retain a number of PCs explaining at least 50% of the total variance and (2) to stop introducing new PCs when the fraction of the explained variance decrease sharply. Once the PC subspace considered, the second step is to detect outliers into this subspace. For the outlier detection, we are calculated the Hotelling’s *T*^2^ statistic (Jackson *et al*. 1991, Villegas *et al*. 2010). The *T*^2^ statistic is distributed according to a *F* distribution and the upper 95% confidence limit of this statistic is determined as the maximal accept limit. In case where outliers are detected, a second application of the sPCA method is required after deleting these ones. The retained PCs are interpreted from the correlation circle and the contribution of individual quantitative variables. Moreover, the quality of representation (squared cosine) is calculated for each variable in order to evaluate which variables are well represented onto different principal plans. Thus, a variable with a contribution superior to the uniform contribution and a high quality of representation has a large impact for the considered PC. The dispersion of sites into principal plan is then illustrated for the variables *PNN* and *NNA* transformed in qualitative variables: the variable *PNN* was divided into 4 groups (low <0.4; medium [0.4; 0.6[; high [0.6; 0.7[; very high ≥0.7) while the variable *NNA* was divided in 3 groups (low <30; medium [30; 50[; high ≥50).

## Supplementary information

Supplementary
