# Supplementary material for: Douglas fir stimulates nitrification in French forest soils
Source: Sci Rep. 2019 Jul 23;9:10687. doi: 10.1038/s41598-019-47042-6 (PMC6650478; doi:10.1038/s41598-019-47042-6)
Supplement: Supplementary file 1 — Supplementary [file 41598_2019_47042_MOESM1_ESM.docx]

**Douglas fir stimulates nitrification in French forest soils**

Bernd Zeller*^1^, Arnaud Legout^1^, Séverine Bienaimé^1^, Bruno Gratia^1,2^, Philippe Santenoise^1^, Pascal Bonnaud^1^, Jacques Ranger^1^

1 INRA Grand-EST Nancy, UR 1138 Biogéochimie des Ecosystèmes Forestiers, Route d'Amance, 54280 CHAMPENOUX, France

2 Office National de Forêts (ONF), Unité Territorial de Darney-Bains, Route des Rochottes, 88260 DARNEY, France

*Corresponding author: Bernd Zeller

Phone: +33 3 83 39 40 77

E-mail: bernhard.zeller@inra.fr

Figure S 1. Map with the distribution of Douglas fir in France (https://inventaire-forestier.ign.fr), location of the 21 experimental sites and former land use (coniferous forest, deciduous forest, farmland).

Table S 1. Spearman’s correlation matrix for soil PNM and PNN, soil properties, vegetation, forest management and past land use. PNM = potential net mineralization rate, PNN = potential net nitrification rate, T0 nitrate = soil nitrate concentration in field fresh samples, SON = soil organic concentration, SOC = soil organic C concentration, C/N Hor A = C/N ratio in the A horizon, Cmic = microbial carbon, Nmic = microbial nitrogen, C/N hor O = C/N of the organic horizon, Ellenberg = Ellenberg N value, GW = G = basal area, TLST = time since last thinning, S = ,
